# Responses of cutaneous C-fiber afferents and spinal microglia after hindlimb cast immobilization in rats

**DOI:** 10.1186/s12576-021-00803-3

**Published:** 2021-06-23

**Authors:** Hiroki Ota, Haruna Takebe, Kazue Mizumura, Toru Taguchi

**Affiliations:** 1 Department of Physical Therapy, Faculty of Rehabilitation, Niigata University of Health and Welfare, 1398 Shimami-cho, Kita-ku, Niigata, 950-3198 Japan; 2 Institute for Human Movement and Medical Science, Niigata University of Health and Welfare, 1398 Shimami-cho, Kita-ku, Niigata, 950-3198 Japan; 3 Department of Physiology, Nihon University School of Dentistry, 1-8-13 Kandasurugadai, Chiyoda-ku, Tokyo, 101-8310 Japan

**Keywords:** Cast immobilization, Hyperalgesia, C-fiber afferents, Single-fiber recording, Spinal microglia, Rats

## Abstract

Previous studies have shown that persistent limb immobilization using a cast increases nociceptive behavior to somatic stimuli in rats. However, the peripheral neural mechanisms of nociception remain unclear. Using single-fiber electrophysiological recordings in vitro, we examined the general characteristics of cutaneous C-fiber afferents in the saphenous nerve and their responsiveness to mechanical and heat stimuli in a rat model of immobilization-induced pain by subjecting the rats to hindlimb cast immobilization for 4 weeks. The mechanical response of C-fibers appeared to increase in the model; however, statistical analysis revealed that neither the response threshold nor the response magnitude was altered. The general characteristics and heat responses of the C-fibers were not altered. The number of microglia and cell diameters significantly increased in the superficial dorsal horn of the lumbar spinal cord. Thus, activated microglia-mediated spinal mechanisms are associated with the induction of nociceptive hypersensitivity in rats after persistent cast immobilization.

## Background

Experimental cast immobilization of the wrist for 4 weeks led to the development of pain during movement in 90% of the participants [1]. Mechanical and cold hyperalgesia, but not heat hyperalgesia, has been reported to occur in the immobilized hands [1]. In rodents, long-term cast immobilization of a limb following fracture and tissue injury leads to nociceptive hypersensitivity similar to that seen in patients with complex regional pain syndrome [2], muscular atrophy [3], osteoporosis [4], and joint contracture [5]. To date, several models of limb immobilization-induced pain have been reported using different induction methods (cast [6–8], waterproof tape [9], wire [10], and suspension of tail/hindlimb [11, 12]), extremities (forelimb [8, 13] and hindlimb [6, 7]), and immobilization periods (2–8 weeks [6, 7, 13, 14]).

Among the various immobilization-induced pain models, the 4-week cast immobilization condition has been reported to result in behavioral nociceptive hypersensitivity to mechanical and thermal stimuli in the ipsilateral hindlimb as the duration of immobilization is prolonged [6, 14]. In the immobilization (IM) model, immunoreactivity for calcitonin gene-related peptide (CGRP), a neurotransmitter expressed in some nociceptive sensory afferents, increased in the superficial dorsal horn of the spinal cord [14]. Ohmichi et al. demonstrated that cast immobilization from the pelvis to the middle of the hind paw for 2-week induced chronic widespread pain, and that the activation of microglia, followed by the activation of astrocytes in the spinal dorsal horn, was involved in the pathological mechanisms associated with immobilization [7, 15]. Moreover, the proportion of neurons in the cervical dorsal horn in response to wrist movement increased in rats that underwent wrist immobilization by cast for 4 weeks [8]. Thus, spinal mechanisms via sensitized neurons and activated glial cells in the dorsal horn could be involved in the pathological mechanisms related to nociceptive hypersensitivity induced by cast immobilization.

Little is known about the nociceptive mechanisms of immobilization-induced pain in the periphery, contrary to that in the central nervous system. In a study, 5 weeks after cast immobilization of the forelimb, the proportion of cervical dorsal root ganglion (DRG) cells with positive immunoreactivity for transient receptor potential vanilloid 1 (TRPV1) and nerve growth factor (NGF) significantly increased [16]. In a model of immobilization-induced pain, CGRP-immunoreactive DRG neurons were found to have a larger cell size on the ipsilateral side of immobilization than on the contralateral side, and the intensity of CGRP immunoreactivity was increased in laminae III–IV but not in laminae I–II [13]. The protein levels of NGF, a neurotrophic factor known to cause hyperalgesia and nociceptor sensitization to mechanical and heat stimuli [17], were increased in the skin and muscle of rats after cast immobilization for 4 weeks [18–20]. Cast immobilization decreases the thickness of the epidermis and increases NGF expression and the density of nerve fibers immunoreactive for P2X_3_ and TRPV1, which are known to be expressed predominantly in small nerve fibers such as unmyelinated C-fibers [6, 20]. In general, the total amount of nociceptive input from the periphery to the spinal cord, which is defined by the multiplication of the number of nociceptive afferents and action potentials induced by a noxious stimulus, increases in hyperalgesic conditions. The former (i.e., increased density of nociceptive afferents in the skin) has been reported in previous studies [6, 20], but the latter (the amount of input from a nociceptive afferent to the spinal cord) remains to be clarified in any immobilization-induced pain model. Thus, in the present study, we examined the general characteristics and response properties of cutaneous C-fiber afferents in rats that underwent hindlimb cast immobilization for 4 weeks.

## Methods

### Animals

Male Sprague–Dawley rats (330–465 g, 11–14 weeks on the day of the experiment; SLC Inc., Japan) were used in this study (*n* = 12 in behavioral experiments, *n* = 9 in electrophysiology experiments, and *n* = 13 rats in immunohistochemistry experiments). Different animals were used in different experiments. Two rats were kept in a cage that was placed on a negative air pressure rack (temperature at 22–24 °C) and exposed to a 12 h light–dark cycle (lights on between 7 a.m. and 7 p.m.). The rats had free access to food and water. All animal experiments were reviewed and approved by the Animal Care and Use Committee of the Niigata University of Health and Welfare and were executed in accordance with the Fundamental Guidelines for Proper Conduct of Animal Experiments and Related Activities in Academic Research Institutions in Japan and the Ethical Guidelines of the International Association for the Study of Pain [21].

### Immobilization-induced pain model

**Fig. 1:**
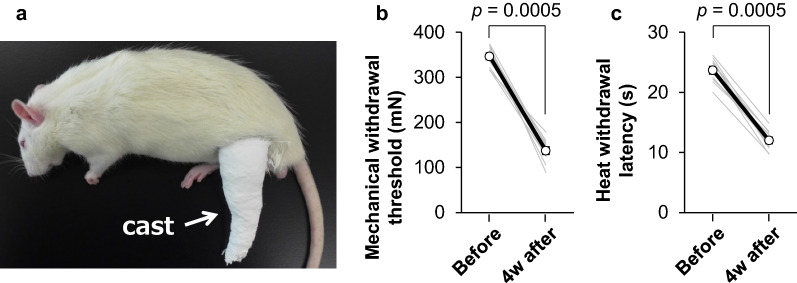
Pain-related behaviors in a rat immobilization-induced pain model. **a** Representative photograph of a rat with cast immobilization of the left hindlimb. **b** Mechanical withdrawal thresholds measured with an electronic von Frey apparatus. **c** Withdrawal latencies to radiant heat as measured using Hargreaves’ apparatus. Mechanical withdrawal thresholds and heat withdrawal latencies were compared before and 4 weeks after cast immobilization using a Wilcoxon matched-pairs signed rank test. Thin gray lines: individual threshold changes (*n* = 12 rats). Thick black line: mean value of the 12 rats with its SEM

We used the same immobilization-induced pain model as previously reported [6]. Rats in the immobilization (IM) group were anesthetized with sodium pentobarbital (40 mg/kg, i.p.). The same anesthetic was supplementally administered as required. Using a plaster cast, the left hindlimb, from the thigh above the knee to the distal foot, was immobilized for 4 weeks with the ankle joint fully plantar flexed (Fig. 1a), while the contralateral (i.e., right) ankle joint remained freely movable. All rats displayed abnormal gait due to limited range of motion in the ankle joint on the side ipsilateral to the immobilization. However, the rats could eat and drink ad libitum in their cages. We checked the condition of the plaster cast as well as the general condition of the rats every one or 2 days. When the cast was slipping or slipped off during the immobilization period, a new cast was wrapped under anesthesia as described above. Special care was taken to prevent tissue damage and disruption of the peripheral circulation, which would cause edema. In the present study, slipping occurred 5–8 times per rat during the 4-week immobilization period. Age-matched rats in the control (CTR) group were treated as those in the IM group, but without cast immobilization.

### Behavioral pain tests

To examine the existence of mechanical nociceptive hypersensitivity after cast immobilization, the mechanical withdrawal threshold was measured before and after the 4-week cast immobilization using an electronic von Frey apparatus, as reported in our previous study [22]. Rats were individually placed in a clear plastic restrainer (21 × 21 × 13 cm^3^) to acclimatize to their environment for 10–30 min before testing. A plantar esthesiometer (tip diameter: 0.5 mm; #37450, Ugo Basile, Italy) was used for withdrawal threshold measurement against punctate mechanical stimuli. The stimulus force was incrementally applied at a rate of 49 mN/s until the rat elicited an escape reaction with a pressure cutoff of 490 mN. The probe of the esthesiometer was applied to the plantar skin on the side ipsilateral to cast immobilization. Measurements were repeated five times, and the threshold for each rat was defined by calculating the average of five measurements. The interstimulus intervals were set to at least 30 s.

To examine whether rats that underwent cast immobilization exhibited thermal hypersensitivity, withdrawal latencies in response to noxious heat were measured following the von Frey test using the same rats. Using the Hargreaves apparatus (390G, IITC, USA), radiant heat was applied to the left plantar skin (the side ipsilateral to cast immobilization) through a glass plate. Measurements were repeated twice at intervals of 1 min, and the mean of the two trials for each rat was used as the latency. The cutoff time was set at 30 s to avoid tissue burn.

Both behavioral tests were performed before and after the 4-week cast immobilization only on the ipsilateral side, since nociceptive behaviors to mechanical and heat stimuli have not been reported to change on the contralateral side in this model [6, 14]. All measurements were obtained during the daytime in a calm room with air-conditioning (22–24 °C).

### Single-fiber electrophysiology

#### Skin–nerve preparations in vitro

To clarify whether nociceptor-mediated peripheral mechanisms were involved in the immobilization-induced pain model, we analyzed the general characteristics and responsiveness of cutaneous C-fiber afferents with an in vitro preparation consisting of the lower hindlimb skin with the saphenous nerve connected [23, 24]. On the day between the second and seventh day after cessation of the 4-week cast immobilization when behavioral nociceptive hypersensitivities were evident, the skin–nerve preparations were harvested from the left hind paw (ipsilateral to cast immobilization) after euthanasia by CO_2_ inhalation. The excised skin was placed in the test chamber and superfused with a modified Krebs–Henseleit solution, which contained 110.9 mM of NaCl, 4.7 mM of KCl, 2.5 mM of CaCl_2_, 1.2 mM of MgSO_4_, 1.2 mM of KH_2_PO_4_, 25 mM of NaHCO_3_, and 20 mM of glucose. The solution was continuously bubbled and saturated with a mixture of O_2_ (95%) and CO_2_ (5%) gas and kept at 32.0 °C ± 0.3 °C at a pH level close to 7.4.

Single C-fiber afferents were searched using the dissection method and identified by manually poking the receptive field (RF) with a blunt glass stick [23]. To measure the conduction velocity (CV) of the afferents, electronic pulses were applied to the RF via a stimulating electrode (frequency of 0.5 Hz, pulse duration of 500 µs, and stimulus intensity of < 50 V). The CV was calculated using the distance between the stimulating electrode placed on the identified RF and the recording electrode, as well as the conduction latency of the action potential. Mechano-sensitive afferent fibers with a CV of less than 2.0 m/s were included for analysis in the present experiment. We did not include low-threshold mechanoreceptors with extremely low von Frey thresholds (approximately < 1 mN) with rapidly adapting mechanical response patterns in this study, since they were assumed to be non-nociceptors. Spontaneous activities of the C-fiber afferents were recorded and analyzed during the observation period of 60 s immediately before the onset of servo-controlled mechanical stimulation. When the C-fibers exhibited at least one action potential during this period, they were considered to be spontaneously active fibers. To quantify the mechanical response of the C-fiber afferents, we used a mechanical stimulator (PS-1S, manufactured by Aizawa S., Goto College of Medical Arts and Science, Tokyo, Japan) with feedback-controlled force regulation that delivered incremental compressive stimulation to the identified RF (up to 294 mN at a constant speed of 9.8 mN/s). The stimulator had a plastic cylindrical probe with a flat circular tip area of 2.28 mm^2^. Following mechanical stimulation, ramped heat stimulation from 32 to 50 °C was applied to the same RF via a small probe (diameter of 1 mm) at a constant rate of 0.6 °C/s using a thermal stimulator that enabled feedback-controlled temperature regulation with a Peltier thermode (Intercross-2000N, Intercross, Co. Ltd., Tokyo, Japan). Two criteria were set to define whether an afferent was sensitive to mechanical and thermal stimuli according to our previous study [23]: (1) the net discharge rate increase during the stimulus period of 30 s was higher than 0.1 imp/s compared to the ongoing discharge rate during the pre-stimulus control period of 60 s, and (2) the instantaneous discharge rate of two consecutive discharges exceeded the mean + 2 SD of the ongoing discharge rate. The response threshold to stimuli was defined as the mechanical stimulus intensity and thermal stimulus temperature that induced a discharge that exceeded the mean frequency + 2 SD of the ongoing discharges during the control period of 60 s, when two or more consecutive discharges exceeded this level. If an afferent fiber elicited no discharge above the response criteria with mechanical stimulation of up to 294 mN, the mechanical threshold was defined to be 294 mN even if it evoked discernible action potentials by manual probing with a blunt glass rod [23].

Action potentials induced by nociceptive stimulation were sorted and analyzed using the DAPSYS data acquisition system (http://www.dapsys.net) [25]. An analog to digital converter (Power Lab 8/35, ADInstruments, New Zealand) was used to store data on nerve activities and analog output from mechanical and thermal stimulators in a computer.

To compare the response patterns of C-fiber afferents between the CTR and IM groups to mechanical and thermal stimuli, we calculated the net evoked discharge rates in each fiber by subtracting the mean spontaneous discharge rates during the 60-s control period immediately before stimulation from the raw discharge rates induced by mechanical or heat stimulation.

### Immunohistochemical labeling of spinal microglia

As the number and morphology of spinal microglia have been shown to change in some pain models [22, 26, 27], and enhanced CGRP expression has been reported in the superficial (but not deep) dorsal horn on the ipsilateral, but not contralateral, side of cast immobilization [6, 14], we measured the number and analyzed the diameter of microglial cells in the dorsal horn on the ipsilateral side. Immunohistochemical staining of spinal microglia was performed as described in our previous study [22]. After 4 weeks of cast immobilization, the rats were deeply anesthetized with sodium pentobarbital (80 mg/kg, i.p.). Phosphate-buffered saline (PBS) followed by fixation (10% formalin) was perfused via the aortic arch. The lumbar segments (L3–L4) were quickly harvested, post-fixed in the same fixative, and cryoprotected in 10% sucrose solution followed by 20% sucrose solution (each for 1 d). Transverse spinal cord sections (thickness of 20 μm) were cut using a cryostat and mounted on a slide. Sections were soaked in PBS containing 1% Triton-X 100, followed by incubation with 5% bovine serum albumin. They were then incubated with rabbit polyclonal antibody against ionized calcium-binding adapter protein 1 (Iba1; 1:1000; 019-19741, Wako) overnight at 4 °C, biotinylated goat anti-rabbit IgG (1:1000; BA-1000, Vector Laboratories) for 60 min at room temperature, and avidin–biotin–peroxidase complex (1:100; PK-6100, Vector Laboratories) for 60 min at room temperature. Iba1 immunoreactivity was visualized by adding 0.003% hydrogen peroxide to PBS containing 0.02% 3,3'-diaminobenzidine tetrahydrochloride and 0.02% nickel ammonium sulfate.

The sections were observed under a light microscope (BZ-X810, Keyence Corp., Japan). Using a software (BZ-H4M, Keyence Corp., Japan), the number and maximum cell diameter of Iba1-immunoreactive (-ir) microglia were counted and measured, respectively, in the dorsal horn laminae I–II, III–IV, and V–VI, which were recognized according to their cytoarchitectonic organization [28]. The investigators responsible for the analysis were blinded to the group to which each section belonged. Two sections were taken for analysis from each animal, and the average number of cells and the diameter of cells were calculated for each animal (*n* = 6 rats in the CTR and *n* = 7 rats in the IM group).

### Statistical analyses

Mechanical withdrawal thresholds and heat withdrawal latencies in behavioral tests, mechanical and heat response patterns of C-fiber afferents during ramp mechanical stimulation in electrophysiological recordings, and the number and diameter of microglial cells in immunohistochemical experiments are expressed as means ± SEMs, while the other parameters of the electrophysiology experiments are expressed as medians with interquartile ranges. Data from the behavioral tests before and after the 4-week cast immobilization were compared using a Wilcoxon matched-pairs signed rank test, since the paired number of samples was relatively small. Electrophysiological data (general characteristics and threshold/magnitude of the responses to mechanical and heat stimuli) between the CTR and IM groups were compared using the Mann–Whitney *U* test, since individual data of C-fiber afferents varied considerably, including those with a value of zero. The proportions of spontaneously active fibers and heat-sensitive fibers between the two groups were compared using Fisher’s exact probability test, since the number of C-fiber afferents analyzed was relatively small. Response patterns were shown as net evoked discharge rates with time to cancel the spontaneous activities of C-fiber afferents, and the patterns during mechanical and heat stimuli were analyzed using a two-way repeated measures ANOVA with Geisser–Greenhouse correction to adjust the degree of freedom, since the sphericity assumption was not met. Sidak’s multiple comparison test was used for the *post-hoc* tests. For immunohistochemistry data, the number and diameter of microglial cells between the CTR and IM groups were compared using the Mann–Whitney *U* test. Statistical significance was set at *p* < 0.05.

## Results

### Pain-related behaviors in an immobilization-induced pain model

Mechanical withdrawal thresholds measured with an electric von Frey apparatus significantly decreased on the ipsilateral side 4 weeks after cast immobilization (*p* = 0.0005, *d* = 5.957, Wilcoxon matched-pairs signed rank test, Fig. 1b). Similarly, withdrawal latencies to noxious radiant heat applied to the paw ipsilateral to the cast immobilization significantly shortened 4 weeks after the onset of immobilization (*p* = 0.0005, *d* = 5.405, Wilcoxon matched-pairs signed rank test, Fig. 1c).

### Single-fiber recordings

**Fig. 2:**
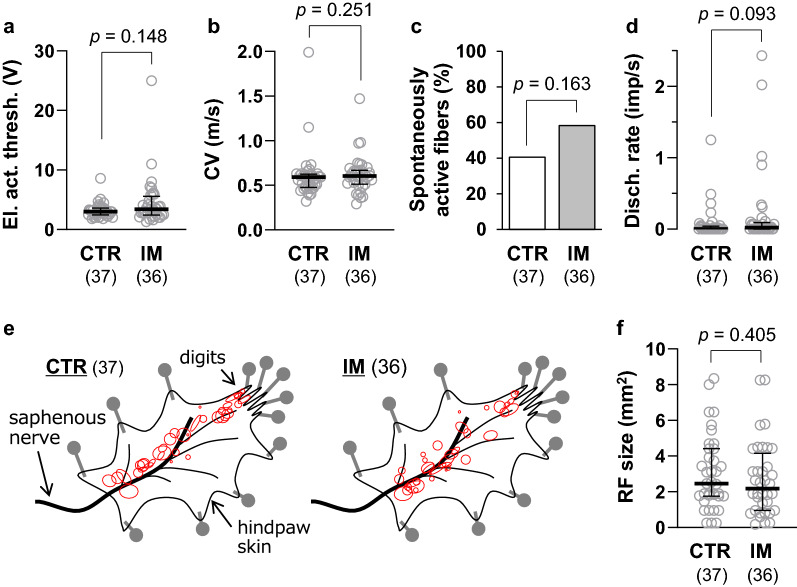
General characteristics of cutaneous C-fiber afferents. **a** Electrical activation threshold. **b** Conduction velocity. **c** Proportion of fibers that exhibit spontaneous (background) activity. **d** Background discharge rate. **e** Distribution of receptive fields (RFs, shown in red). **f** Size of RFs. In **a**, **b**, **d**, and **f**, individual data are plotted as gray circles and summarized data are represented as medians with interquartile ranges (black lines). Note that there were no significant differences between the CTR (*n* = 37) and IM (*n* = 36) groups in any of the general characteristic measures (Mann–Whitney *U* test for **a**, **b**,** d** and **f**, and Fisher’s exact probability test for **c**)

A total of 73 C-fibers were recorded and analyzed (*n* = 37 fibers from four CTR rats and *n* = 36 fibers from five IM rats). The general characteristics of the C-fibers are shown in Fig. 2. Median electrical activation thresholds, which were measured by applying electrical stimulus to an RF via a stimulating electrode, were 3.0 V in the CTR and 3.4 V in the IM group, and the thresholds did not differ between the two groups (*p* = 0.148, *d* = 0.438, Mann–Whitney *U* test, Fig. 2a). Median CVs of C-fiber afferents were 0.59 m/s in the CTR and 0.61 m/s in the IM group, and they did not differ between the two groups (*p* = 0.251, *d* = 0.055, Mann–Whitney *U* test, Fig. 2b). The proportions of spontaneously active fibers, which exhibited one or more discharges during the observation period of 60 s without any intentional stimuli, was 41% in the CTR group and 58% in the IM group, and the proportions did not differ between the two groups (*p* = 0.163, odds ratio = 0.487, Fisher’s exact probability test, Fig. 2c). The median spontaneous discharge rates were low at 0 imp/s in the CTR group and 0.02 imp/s in the IM group. The discharge rates did not differ between the two groups (*p* = 0.093, *d* = 0.345, Mann–Whitney *U* test, Fig. 2d). As shown in Fig. 2e, the RFs were located along the main saphenous nerve branch from the entry zone to the digits. The distribution patterns were similar between the CTR and IM groups and were similar to those reported in our previous study [23]. The median RF sizes were 2.5 mm^2^ in the CTR group and 2.2 mm^2^ in the IM group, and the sizes did not differ between the two groups (*p* = 0.405, *d* = 0.172, Mann–Whitney *U* test, Fig. 2f).

**Fig. 3:**
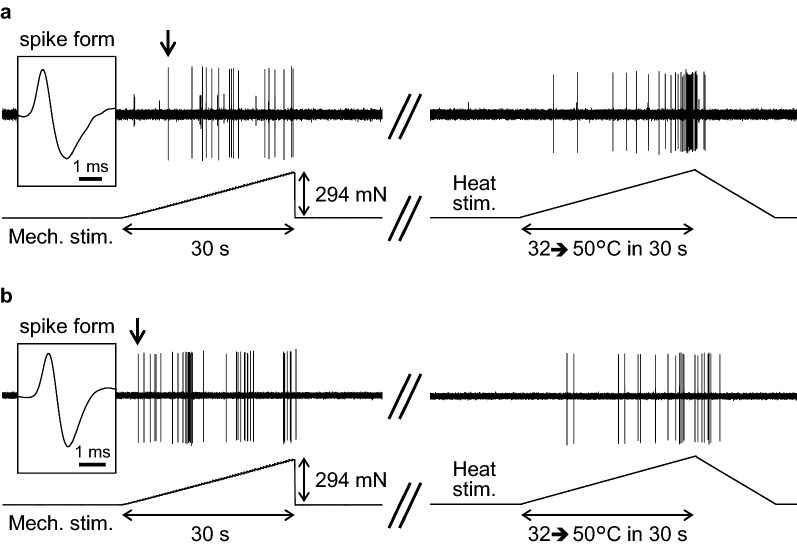
Mechanical and heat responses of cutaneous C-fiber afferents. **a** Representative recording of a C-fiber obtained from the CTR group. **b** Recording from the IM group. Stimulus outputs of mechanical (294 mN in 30 s) and heat stimuli (from 32 to 50 °C in 30 s) are shown under the original recordings. An inset on the left side of each recording indicates the shape of the individual spike indicated by an arrow. Recordings of mechanical and heat responses are continuous and separated by a double slash (//) at a time interval of 5–10 min

**Fig. 4:**
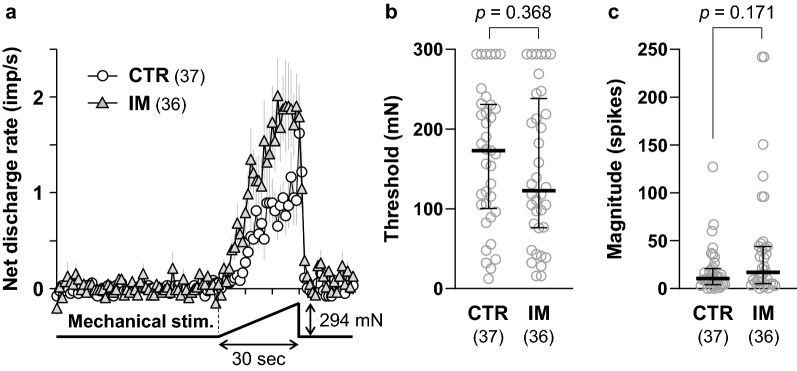
Mechanical response of cutaneous C-fiber afferents. **a** Response patterns to a ramped mechanical stimulus (294 mN in 30 s). Each data point is represented by the mean discharge rate ± its SEM (gray lines). Two-way repeated measures ANOVA with Geisser–Greenhouse correction revealed an insignificant effect of GROUP (*F*_(1, 71)_ = 3.424, *p* = 0.068, partial eta squared (*η*^2^) = 0.082) but significant effects of TIME (*F*_(4.790, 340.1)_ = 12.82, *p* < 0.0001, partial *η*^2^ = 0.928) as well as GROUP x TIME interaction (*F*_(30, 2130)_ = 1.573, *p* = 0.025, partial *η*^2^ = 0.752). *Post-hoc* test detected no significant difference in the discharge rate at any timepoint during the stimulation period of 30 s (31 data points every 1 s) between the CTR and the IM groups. **b** Mechanical response threshold, as measured using a servo-controlled mechanical stimulator. **c** Response magnitude (total net evoked spikes during the stimulation). No significant differences were observed in the response threshold and magnitude between the CTR (*n* = 37) and IM (*n* = 36) groups (Mann–Whitney *U* test for **b** and **c**)

Figure 3 shows the responses of a representative C-fiber to mechanical and heat stimuli in the CTR (Fig. 3a) and IM groups (Fig. 3b). The overall responsiveness to noxious stimuli was not significantly different between the two groups, although there appeared to be a trend in which the mechanical response was greater (lower threshold and higher number of action potentials) in the IM group than in the CTR group. The average mechanical response patterns of all the C-fibers recorded in the two groups are shown in Fig. 4a. The discharge rate appeared to be higher with a steeper build-up of the response in the IM group than in the CTR group. However, when net evoked discharge rates, which were calculated by subtracting the mean spontaneous discharge rates during the 60-s control period immediately before stimulation from the raw discharge rates at each timepoint during mechanical stimulation, were analyzed with two-way repeated measures ANOVA with Geisser–Greenhouse correction, an insignificant effect of GROUP (*F*_(1, 71)_ = 3.424, *p* = 0.068, partial eta squared (*η*^2^) = 0.082) and significant effects of TIME (*F*_(4.790, 340.1)_ = 12.82, *p* < 0.0001, partial *η*^2^ = 0.928) as well as GROUP × TIME interaction (*F*_(30, 2130)_ = 1.573, *p* = 0.025, partial *η*^2^ = 0.752) were detected. *Post-hoc* tests detected no significant difference in the discharge rate at any timepoint during the stimulation period of 30 s (31 data points every 1 s) between the CTR and IM groups (Fig. 4a). Accordingly, the mechanical response threshold (*p* = 0.368, *d* = 0.199, Mann–Whitney *U* test, Fig. 4b) and the magnitude (total net evoked spikes) did not differ between the CTR and IM groups (*p* = 0.171, *d* = 0.491, Mann–Whitney *U* test, Fig. 4c).

**Fig. 5:**
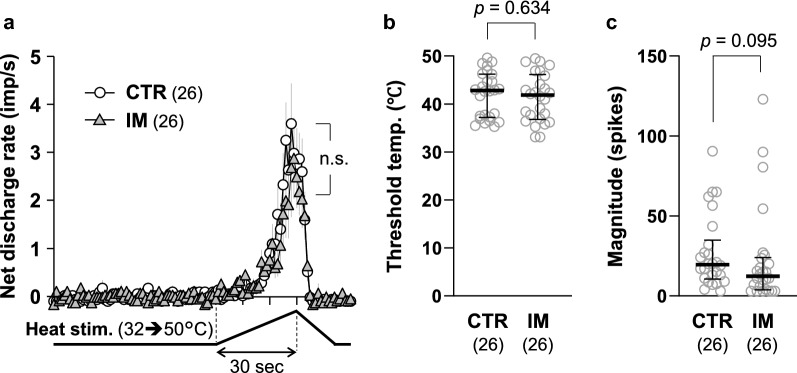
Heat response of cutaneous afferents. **a** Response patterns of heat-sensitive C-fiber afferents to a ramped heat stimulus (from 32 to 50 °C in 30 s). Each data point is represented by the mean discharge rate ± its SEM (gray lines). Two-way repeated measures ANOVA with Geisser–Greenhouse correction revealed a significant effect of TIME (*F*_(2.993, 149.7)_ = 16.21, *p* < 0.0001, partial *η*^2^ = 0.387), but insignificant effects of GROUP (*F*_(1, 50)_ = 0.304, *p* = 0.584, partial *η*^2^ = 0.012) and GROUP x TIME interaction (*F*_(30, 1500)_ = 0.665, *p* = 0.916, partial *η*^2^ = 0.027). **b.** Threshold temperature to heat stimulation. **c** Response magnitude (net evoked spikes). No significant differences were observed in the response threshold and magnitude between the CTR (n = 26) and IM (n = 26) groups (Mann–Whitney *U* test for **b** and **c**)

The proportion of heat-sensitive C-fibers was 70.3% (26 of 37 fibers) in the CTR group and 72.2% (26 of 36 fibers) in the IM group, which did not differ significantly between the two groups (*p* > 0.9999, odds ratio = 0.909, Fisher’s exact probability test). The discharge rates of heat-sensitive C-fibers increased as the stimulus temperature increased, and the response patterns were similar in the CTR and IM groups (Fig. 5a). Two-way repeated measures ANOVA with Geisser–Greenhouse correction revealed a significant effect of TIME (*F*_(2.993, 149.7)_ = 16.21, *p* < 0.0001, partial *η*^2^ = 0.387), but insignificant effects of GROUP (*F*_(1, 50)_ = 0.304, *p* = 0.584, partial *η*^2^ = 0.012) and GROUP × TIME interaction [*F*_(30, 1500)_ = 0.665, *p* = 0.916, partial *η*^2^ = 0.027]. Heat response thresholds were 42.8 °C in the CTR group and 41.9 °C in the IM group, and the threshold temperature did not differ significantly between the two groups (*p* = 0.634, *d* = 0.123, Mann–Whitney *U* test, Fig. 5b). The magnitudes of the heat response (net evoked spikes) did not significantly differ between the two groups (*p* = 0.095, *d* = 0.141, Mann–Whitney *U* test, Fig. 5c).

### Spinal microglia

**Fig. 6:**
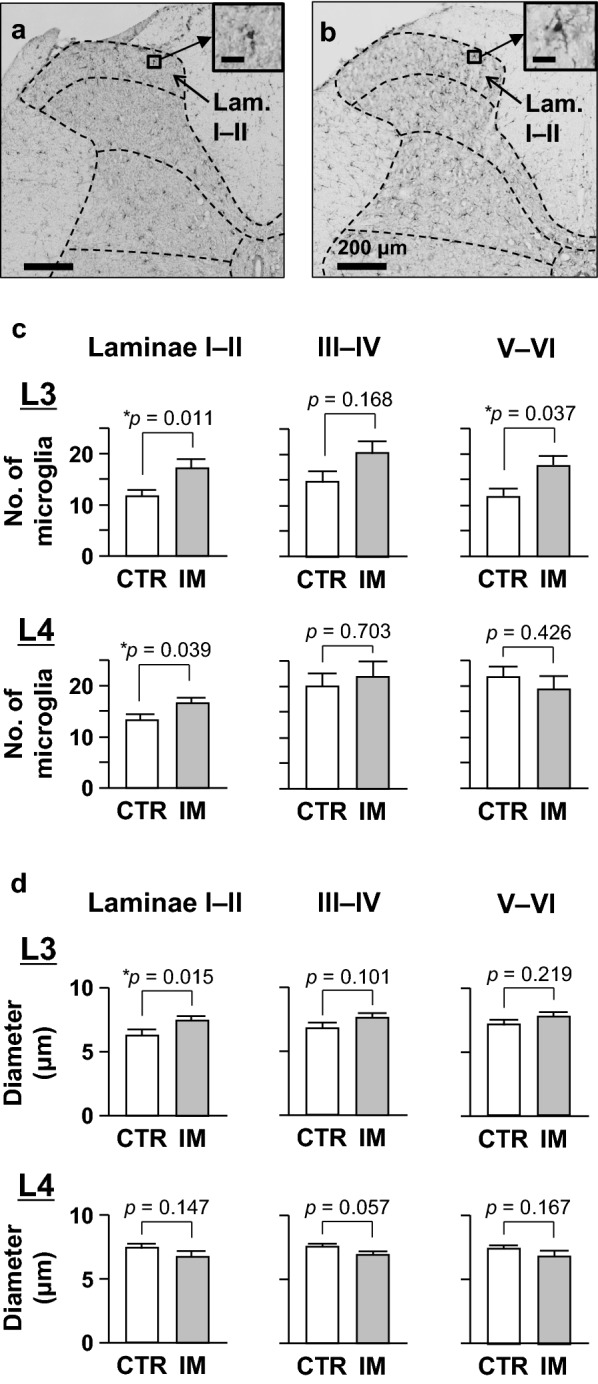
Immunohistochemical labeling of microglia in the spinal dorsal horn. **a** Iba1-immunoreactive microglial cells on the side ipsilateral to the cast immobilization at spinal segment L3 in the CTR group. **b** Those in the IM group. Scale bars in the insets = 20 µm. **c** Number of microglial cells in laminae I–II, III–IV, and V–VI on the side ipsilateral to the immobilization at segments L3 and L4. **d** Cell diameters of the same cells counted. The numbers and diameters were compared between the CTR (*n* = 6) and the IM groups (*n* = 7) using Mann–Whitney *U* test

Representative photomicrographs of the spinal segment L3 of the CTR and IM groups are shown in Fig. 6a, b, respectively. To examine any alterations in the number and morphology of spinal microglia, we counted and measured the diameters of all Iba1-positive microglial cells in the dorsal horn ipsilateral to the cast immobilization.

The number of microglia in laminae I–II was significantly higher in the IM group than in the CTR group at the L3 and L4 spinal segments (*p* = 0.011, *d* = 1.538 for L3 and *p* = 0.039, *d* = 1.462 for L4, Mann–Whitney *U* test, Fig. 6c). The number of laminae III–IV did not differ between the two groups at L3 and L4 (*p* = 0.168, *d* = 1.060 for L3 and *p* = 0.703, *d* = 0.262 for L4). The number of laminae V–VI was significantly higher in the IM group than in the CTR group at L3 (*p* = 0.037, *d* = 1.411), but not at L4 (*p* = 0.426, *d* = 0.416).

The cell diameter in laminae I–II was significantly larger in the IM group than in the CTR group at spinal segment L3 (*p* = 0.015, *d* = 1.440, Mann–Whitney *U* test, Fig. 6d), but not at L4 (*p* = 0.147, *d* = 0.803). The diameters of laminae III–IV did not differ between the two groups at L3 (*p* = 0.101, *d* = 1.041) and L4 (*p* = 0.057, *d* = 1.241). The diameters of laminae V–VI did not differ between the two groups at L3 (*p* = 0.219, *d* = 0.793) and L4 (*p* = 0.167, *d* = 0.708).

## Discussion

### Peripheral mechanisms of immobilization-induced pain

In the present study, mechanical responses of cutaneous C-fiber afferents did not change in the saphenous nerve of rats that underwent 4-week hindlimb immobilization, whereas behavioral nociceptive hypersensitivity to mechanical stimuli was evident. We detected only a statistically significant GROUP × TIME interaction in the mechanical response patterns; however, the effect of GROUP was insignificant, and *a post-hoc* test detected no significant difference in the discharge rate during the stimulation period of 30 s between the CTR and IM groups. In addition, neither the threshold nor the magnitude of the mechanical response differed between the CTR and IM groups. Concerning the heat responses, we detected no changes between the two groups, whereas behavioral nociceptive hypersensitivity to heat was obvious in the immobilization-induced pain model.

Recent studies have reported that the expression of NGF proteins is upregulated in the plantar (glabrous) skin and in the gastrocnemius muscle of rats that have undergone hindlimb cast immobilization for 4 weeks [18, 19]. NGF is known to induce potent sensitization of nociceptors to mechanical and heat stimuli, resulting in mechanical and heat hyperalgesia [17]. While the reason behind these discrepancies is unclear, it is possible that immobilization-induced upregulation of NGF may not be sufficient to induce nociceptor sensitization. Another explanation may be that the RF was located on the dorsal (hairy) surface of the foot in the electrophysiological experiment, but the von Frey test was performed on the plantar skin.

Afferent activities of the medial articular nerve are reported to increase in response to passive movement of the knee in rats that underwent knee joint immobilization for 6 weeks using wire and an aluminum splint [29]. The differences between that study and ours may be due to differences in the induction methods used, the duration of immobilization, and/or the afferents examined (i.e., skin vs. joint). In general, the intensity of nociceptive signals can be determined by the number of recruited nociceptive fibers and their firing frequency. In the immobilization model used in this study, the expression of NGF and the density of P2X_3_- and TRPV1-positive nerve fibers is reported to increase in the epidermis as the immobilization period is prolonged [20]. As the responses of cutaneous C-fiber afferents to mechanical and heat stimuli remained unchanged in our study, an increased density of nociceptive fibers in the epidermis could be responsible for the hyperalgesic nociceptive behaviors. Another possibility is that nociceptive afferents in peripheral branches other than the saphenous nerve, such as the sural, tibial, and peroneal nerves innervating the hindlimb skin, are sensitized in the immobilization-induced pain model used in this study.

There were discrepancies between pain-related behaviors and afferent activities in the present study. Such discrepancies have been reported in rats with advancing age [23] and in a rat reserpine-induced fibromyalgia model [22]. In aged Sprague–Dawley rats (130 weeks), mechanical withdrawal thresholds remain unchanged, although peripheral mechanical inputs from cutaneous C-fiber afferents are dramatically reduced [23]. Withdrawal latencies to noxious heat stimuli are shortened in aged rats, while the heat responses of C-fiber afferents remain unchanged [23]. In a rat reserpine-induced fibromyalgia model, in which rats exhibit lower locomotive activities (hypokinesia) for a couple of days after reserpine treatment, nociceptive behaviors to mechanical and heat stimuli are enhanced [22, 30]. Accordingly, mechanical responses of mechanosensitive C-fiber afferents are facilitated, but the proportion of mechanosensitive C-fiber afferents among all C-fibers is paradoxically decreased. Responses to noxious heat via C-fiber afferents in rats of the reserpine-induced fibromyalgia model remain unchanged [22]. These discrepancies between behavioral outcomes and afferent activities may occur in retrogressive diseases associated with physical inactivity (i.e., disuse) and aging. In particular, central mechanisms acting via sensitized neurons and activated glial cells might explain these discrepancies.

### Spinal mechanisms of immobilization-induced pain

In the present study, an increased number and diameter of microglial cells were observed in the lumbar segments, especially in the dorsal horn laminae I–II. We found a significant increase in the cell number in laminae V–VI at segment L3. No significant changes in the number and diameter of cells were detected in the other dorsal horn laminae. According to a rat dermatome map [31], the skin area covered by the plaster cast used in our IM model is innervated by afferent fibers projecting to the L2–L5 lumbar segments. Microglial activation in the spinal cord is known to play critical roles in several pain models associated with inflammation, nerve injury, and fibromyalgia [22, 32–34], with their activation characterized by an increased number and size of the cells. Thus, spinal mechanisms via activated glial cells in the dorsal horn could be involved in the pathological mechanisms related to nociceptive hypersensitivity induced by hindlimb cast immobilization.

In the IM model used in this study, CGRP immunoreactivity has been reported to increase in laminae I–II of the spinal dorsal horn [14], which is where peripheral nociceptive inputs from Aδ- and C-fibers are primarily processed [35]. On analyzing spinal cord slices, the postsynaptic action of CGRP was shown to induce synaptic plasticity in the substantia gelatinosa neurons of a rat model of joint arthritis [36]. CGRP released in the spinal cord in response to peripheral input is thought to sensitize second-order neurons by increasing their sensitivity to the excitatory neurotransmitter glutamate [37] and by triggering the production and release of inflammatory mediators and pro-nociceptive cytokines from the microglia and astrocytes. Taken together, spinal mechanisms acting via sensitized neurons and activated glial cells in the dorsal horn could be pathological mechanisms underlying the nociceptive hypersensitivity induced by persistent cast immobilization.

## Study limitations

In the behavioral tests done in the present study, it was not possible to blind the experimenter to the experimental groups, because the immobilized rats had limited ankle joint ranges of motion owing to persistent immobilization.

Another limitation is that pain-related behaviors and pathological mechanisms associated with limb immobilization might differ across immobilization models [6–14]. As shown in Fig. 1a, the IM pain model used in the present study was developed by immobilizing the hindlimb, from the thigh above the knee to the distal foot, for 4 weeks [6, 14]. In the absence of visible circulatory disturbances, such as edema and necrosis of the immobilized skin, behavioral nociceptive hypersensitivity to mechanical and heat stimuli worsens in the hind paw on the ipsilateral but not contralateral side of immobilization as the duration of immobilization is prolonged. On the contrary, Ohmichi et al. reported that unilateral cast immobilization from the pelvis to the middle of the hindpaw for 2 weeks results in widespread pain in the bilateral hind paws and tail and that the pain persists for 5–10 weeks after cast removal [7]. Nociceptive behavioral hypersensitivities are associated with robust inflammation in the immobilized hindlimb due to ischemia and reperfusion injury, which starts 2 h after cast removal and continues for another week. Thus, this model is referred to as the chronic post-cast pain (CPCP) model [7] and is distinct from the IM model. The difference between the two models may result from the different immobilization periods and areas covered by a plaster cast, and/or from the fact that a plaster cast needs to be rewrapped in the IM model, while a cast does not need to be rewrapped in the CPCP model. Frequent rewrapping of the plaster cast at short time intervals in the IM model can minimize the robust inflammatory changes induced by oxidative stress due to ischemia and reperfusion injury, which is evident in the CPCP model [6, 7, 14].

The last limitation of this study was that we only examined changes in the unmyelinated C-fibers of the saphenous nerve branch. Possible contributions of C-fiber low-threshold mechanoreceptors and myelinated Aδ-fibers in the saphenous branch, as well as other nerve branches, have not been investigated in the present study.

## Conclusions

Four-week hindlimb cast immobilization increased nociceptive behaviors to mechanical and thermal stimuli in rats. The mechanical response of cutaneous C-fibers appeared to increase in the model; however, statistical analysis revealed that neither the response threshold nor the response magnitude to thermal stimuli was altered. Microglial activation was evident, especially in the superficial dorsal horn, on the ipsilateral side of the limb that underwent cast immobilization. These results might be associated with induction of nociceptive hypersensitivity in the rat immobilization-induced pain model used in the present study.

## Data Availability

The datasets used and/or analysed during the current study are available from the corresponding author on reasonable request.

## Abbreviations

CGRP: Calcitonin gene-related peptide
CTR: Control
CV: Conduction velocity
DRG: Dorsal root ganglion
Iba1: Ionized calcium-binding adapter protein 1
IgG: Immunoglobulin G
IM: Immobilization
-ir: -Immunoreactive
NGF: Nerve growth factor
PBS: Phosphate buffered saline
RF: Receptive field
TRPV1: Transient receptor potential vanilloid 1
